# The use of fluorescence angiography to assess bowel viability in the acute setting: an international, multi-centre case series

**DOI:** 10.1007/s00464-022-09136-7

**Published:** 2022-02-23

**Authors:** Johanna J. Joosten, Grégoire Longchamp, Mohammad F. Khan, Wytze Lameris, Mark I. van Berge Henegouwen, Wilhelmus A. Bemelman, Ronan A. Cahill, Roel Hompes, Frédéric Ris

**Affiliations:** 1 Department of Surgery, Amsterdam University Medical Centres (UMC), University of Amsterdam, Cancer Centre Amsterdam, Meibergdreef 9, Amsterdam, the Netherlands; 2 Division of Digestive Surgery, University Hospitals of Geneva, 1205 Geneva, Switzerland; 3 Department of Surgery, Mater Misericordiae University, Hospital, 47 Eccles Street, Dublin 7, Ireland; 4 UCD Centre for Precision Surgery, University College Dublin, Dublin, Ireland

**Keywords:** Fluorescence angiography, Acute setting, Change of management, Ischaemia, Case series

## Abstract

**Introduction:**

Assessing bowel viability can be challenging during acute surgical procedures, especially regarding mesenteric ischaemia. Intraoperative fluorescence angiography (FA) may be a valuable tool for the surgeon to determine whether bowel resection is necessary and to define the most appropriate resection margins. The aim of this study is to report on FA use in the acute setting and to judge its impact on intraoperative decision making.

**Materials and methods:**

This is a multi-centre, retrospective case series of patients undergoing emergency abdominal surgery between February 2016 and 2021 in three general/colorectal units where intraoperative FA was performed to assess bowel viability. Primary endpoint was change of management after the FA assessment.

**Results:**

A total of 93 patients (50 males, 66.6 ± 19.2 years, ASA score ≥ III in 85%) were identified and studied. Initial surgical approach was laparotomy in 66 (71%) patients and laparoscopy in 27 (29% and seven, 26% conversions). The most common aetiologies were mesenteric ischaemia (*n* = 42, 45%) and adhesional/herniae-related strangulation (*n* = 41, 44%). In 50 patients a bowel resection was performed. Overall rates of anastomosis after resection, reoperation and 30-day mortality were 48% (*n* = 24/50, one leak), 12% and 18%, respectively. FA changed management in 27 (29%) patients. In four patients (4% overall), resection was avoided and in 21 (23%) extra bowel length was preserved (median 50 cm of bowel saved, IQR 28–98) although three patients developed further ischaemia. FA prompted extended resection (median of 20 cm, IQR 10–50 extra bowel) in six (6%) patients.

**Conclusion:**

Intraoperative use of FA impacts surgical decisions regarding bowel resection for intestinal ischaemia, potentially enabling bowel preservation in approximately one out of four patients. Prospective studies are needed to optimize the best use of this technology for this indication and to determine standards for the interpretation of FA images and the potential subsequent need for second-look surgeries.

**Supplementary Information:**

The online version contains supplementary material available at 10.1007/s00464-022-09136-7.

Intestinal ischaemia develops as a consequence of severe hypoperfusion caused by a variety of reasons which, if left untreated, leads to transmural necrosis of the bowel wall followed by perforation, peritonitis, sepsis and organ failure. Even with treatment, this cascade results in high mortality rates exceeding 60% [1–3]. Patient's survival depends on prompt recognition and treatment to either achieve revascularization before ischaemia progresses to intestinal gangrene or resection of ischaemic segments of bowel. The incidence of treatable intestinal ischaemia appears to be rising, partly due to an increased awareness among clinicians but also an increasing incidence due to an ageing population surviving with severe cardiovascular or systemic disease [4].

At operation, determination of adequacy of bowel perfusion is essential and, where frank ischaemia is present, judgement of resection margins is vital. Extensive resections should be carefully considered, as removal of large segments of small bowel can result in short bowel syndrome (SBS) with intestinal failure. This is associated with poor quality of life and significant morbidity that increases with age [5]. If, however, the surgical approach is too conservative, and ischaemic bowel is left in situ, further clinical deterioration may result needing reoperation and increasing the risk of death. In the acute setting, judging the most appropriate resection extent may be difficult as a wide range of variables including haemodynamic instability and vasopressor support may co-exist. Also surgeon experience may be important [6]. Although many tools for intraoperative intestinal perfusion assessment have been considered over the years [7, 8], none have become standard due to their complexity and difficulty in reproducibility as well as their cost. A straight forward and useful intraoperative test would be very helpful.

Intraoperative, real time fluorescence angiography (FA) is a promising technique that has shown value for evaluation of adequate perfusion in gastrointestinal anastomoses in the elective setting [9, 10]. Unfortunately, little is known about the application of FA in the acute setting [11]. Therefore, we aimed to report the impact of the use of FA in the acute setting on intraoperative decisions and clinical outcomes in three academic centres.

## Materials and methods

We performed a retrospective analysis of a non-consecutive case series of all patients undergoing emergency surgery for bowel ischaemia in which FA was performed between February 2016 and 2021 in three tertiary referral centres. This study has been approved by the medical ethical committee of the Amsterdam University Medical Centres (AUMC)—location Amsterdam Medical Centre and has therefore been performed in accordance with the ethical standards laid down in the 1964 Declaration of Helsinki and its later amendments. This case series has been reported in line with the PROCESS Guideline [12]. Inclusion criteria were patients over 18 years old that underwent FA using indocyanine green (ICG) to assess intestinal perfusion during an emergency procedure for suspicion of bowel ischaemia. Patients were excluded when FA was performed after intestinal resection or with the sole purpose to assess perfusion of an anastomosis. Patient data with baseline characteristics, operative details and postoperative outcomes were retrospectively collected from the prospective maintained electronic patient systems of the different hospitals.

### FA procedure and endpoints

All patients underwent acute surgical exploration, either via laparoscopy or laparotomy. In the case of mesenteric ischaemia, revascularization (if needed) was performed prior to fluorescence assessment. In all, intestinal perfusion appeared compromised (by macroscopical colourization of the bowel) and firstly the surgeon assessed the compromised segment of bowel by visual examination and determined a possible surgical plan; i.e. if bowel resection was necessary and if so the resection margins, subsequently FA assessment was performed by intravenous injection of a single bolus of ICG (Verdye, Diagnostic Green, Aschheim-Dornach, Germany). The definitive surgical plan based on the FA was then determined and carried out. Change of management due to FA(CoM-FA) was defined as any deviation from the initial strategy determined by visual examination according to the result of the FA assessment. FA was performed with Stryker (Stryker, Kalamazoo, MI, USA.) imaging systems: either PINPOINT laparoscopic imaging system, Stryker 1688, or SPY Portable Handheld Imager (SPY-PHI). The primary outcome was CoM-FA. Secondary outcomes included length (in cm) of additional bowel preserved or resected after FA, need for and number of reoperations, and mortality within 30 days. Reoperations were divided into planned (second look) and unplanned reoperations.

### Statistics

All analyses were executed in IBM SPSS version 26 (IBM Corp. in Armonk, NY). The Shapiro–Wilk normality test was used to assess normal distribution. Data are expressed as mean and standard deviation (SD) for normally distributed continuous variables, median and interquartile range for non-normally distributed variables and proportions for binary variables.

## Results

### Patient characteristics

In total, 93 patients were included in the study with a mean age of 66.6 years at time of surgery (SD 19.2) and an American Society of Anaesthesiologists (ASA) score greater than two in 85%. Of all patients, 50 (54%) were male. The patient characteristics are outlined in Table 1. The interventions were performed by 38 different surgeons with various level of surgical experience and more than half of the surgeries (*n* = 52/93) were performed by senior surgical trainees.

**Table 1:** Patient characteristics

	Total	CoM-FA	No CoM-FA
Total number of patients	93	27	66
Male	50 (54%)	15 (56%)	35 (53%)
Female	43 (46%)	12 (44%)	31 (47%)
Age in years, mean ± SD	66.6 ± 19.2	64.3 ± 20.3	67.6 ± 18.8
ASA score			
I	0	0	0
II	5 (5%)	2 (7%)	12 (18%)
III	53 (57%)	13 (48%)	40 (61%)
IV	26 (28%)	12 (44%)	14 (21%)
Mean BMI, (kg/m^2^) ± SD	25.2 ± 5.0	25.1 ± 4.4	25.2 ± 5.2
Cardiovascular history	32 (34%)	10 (37%)	22 (33%)
Diabetes	22 (24%)	10 (37%)	12 (18%)

*CoM-FA* change of management due to fluorescence angiography, *ASA* American Society of Anaesthesiology, *BMI* Body Mass Index

### Operation characteristics

In the majority of cases (*n* = 66/93, 71%), a laparotomy was performed. The remainder had their surgery commenced with laparoscopy with seven then needing conversion to laparotomy (26% conversion rate). Of laparotomies, 52% (*n* = 34/66) were performed by consultants and 48% (*n* = 32/66) by senior trainees while for laparoscopy the proportions were 35% (*n* = 7/20) and 65% (*n* = 13/20), respectively. All laparoscopic procedures (*n* = 7) which required conversion were carried out by senior trainees. The most common underlying aetiologies of ischaemia were mesenteric ischaemia in 45% (*n* = 42/93) of patients and strangulation due to adhesion or internal herniation in 44% (*n* = 41/93). 4% (*n* = 4/93) were caused by a volvulus while the last 6% (*n* = 6/93) concerned other causes: such as occlusive tumour and perforation. In 50 out of 93 patients, bowel resection was carried out (12 colonic, 24 small bowel and 14 both colonic and small bowel). Among them, 48% (*n* = 24/50) had an anastomosis constructed.

### CoM-FA

**Fig. 1:**
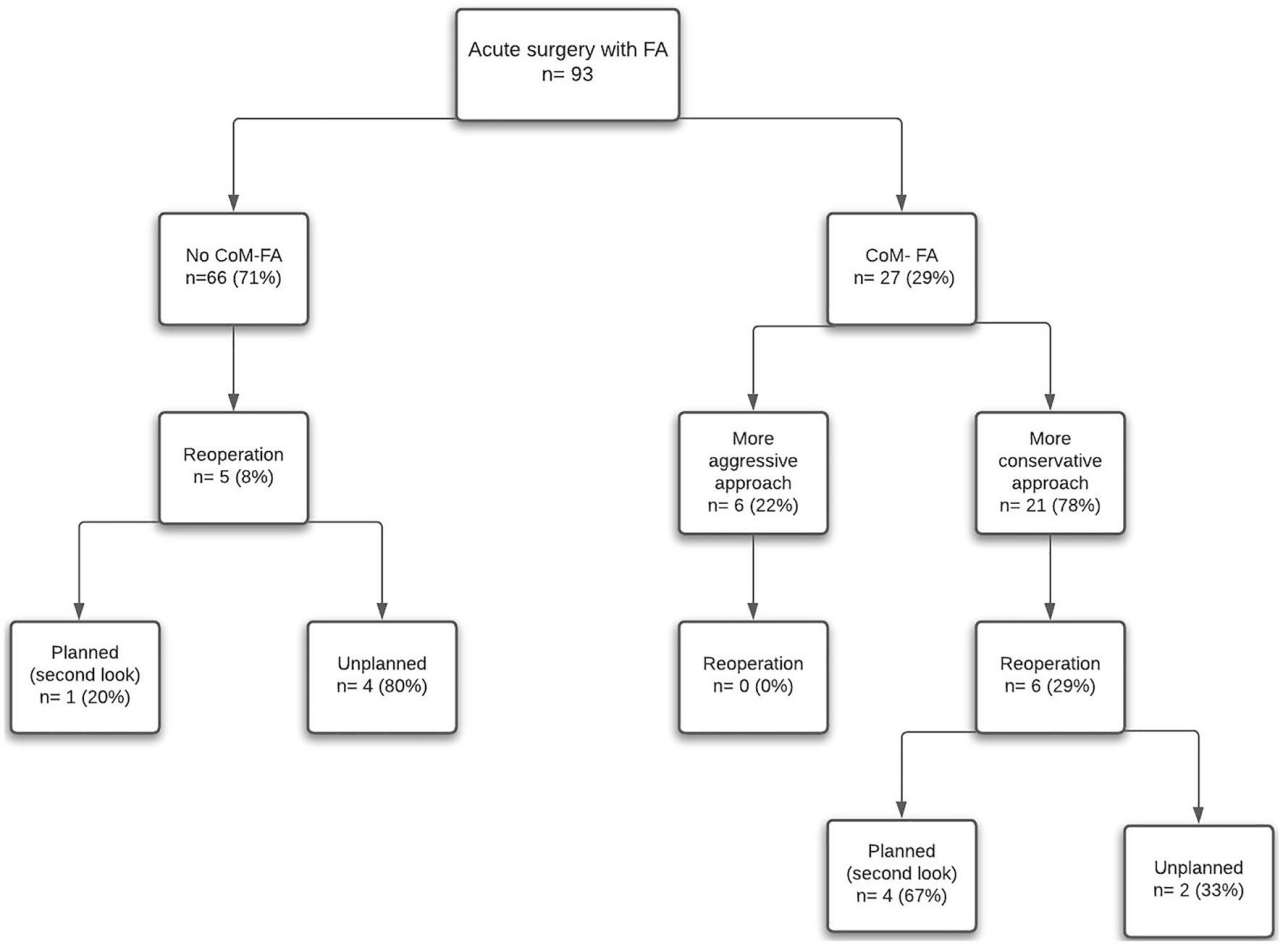
Represents a flowchart of all patients with and without a change of management due to FA(CoM-FA/no CoM-FA) and the reoperation rates of the groups

**Fig. 2:**
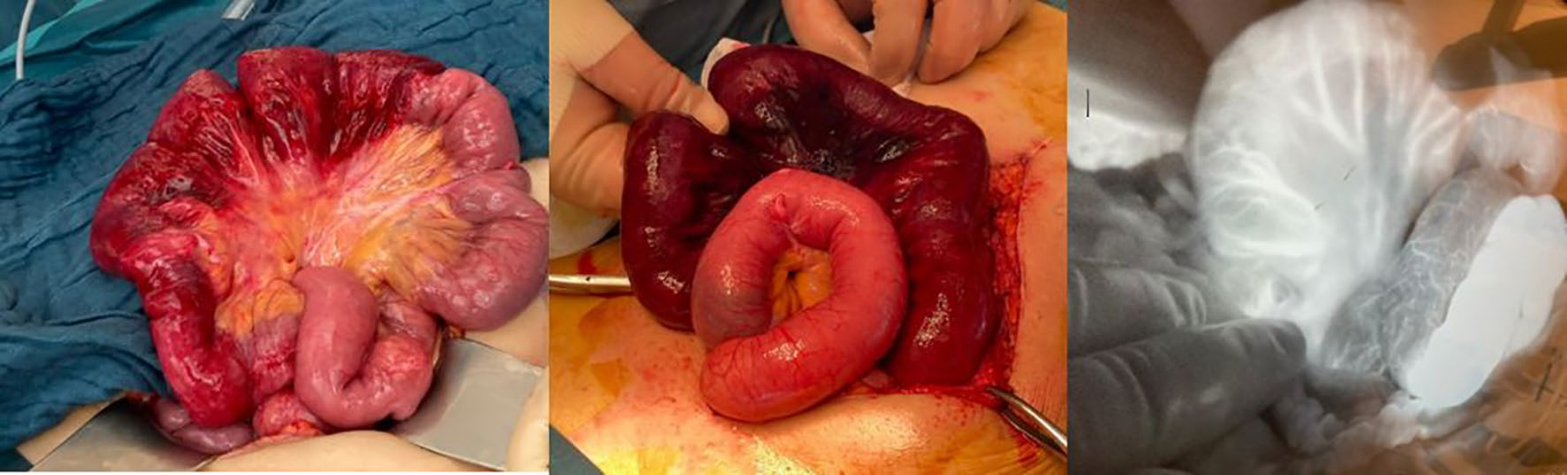
Represents a case in which the perfusion was compromised in visual examination. FA assessment showed clear fluorescence enhancement, no resection was carried out. The patient had an uncomplicated postoperative course

FA resulted in a CoM in 29% of patients (*n* = 27/93). CoM-FA led to either a more conservative or a more aggressive approach. The patient characteristics between these two groups are portrayed in Table 1 and outcomes overall as well as by aetiologies and COM in Table 2. Change to a more conservative approach occurred in 21 patients (Fig. 1, Table 2); in four of these patients (*n* = 4/21, 19%), resection was avoided (Fig. 2) and in the remaining 17 (81%), FA led to resection of a shorter segment of bowel. In this group, CoM-FA supporting a more conservative approach, a median of 50 cm of bowel (IQR 28–98) was preserved (for more detailed overview see Supplementary Table 1). In the CoM-FA group, FA assessment resulted in a more aggressive approach in six patients (n = 6/27), leading to a median of 20 cm (IQR 10–50) of additional resected bowel. The seniority of the surgeons was not decisive in the frequency of altering the surgical plan after FA; 52% of the cases in CoM-FA were by a senior surgeons versus 48% by a senior surgical trainee.

**Table 2:** Outcomes by ischaemia aetiology and whether the fluorescence assessment changed management or not

Outcome	No change of management	Change of management		Total
		More conservative approach	More aggressive approach	
	66 (63%)	21 (23%)	6 (6%)	93 (100%)
Aetiology ischaemia				
Mesenteric ischaemia	25 (38%)	12 (57%)	5 (83%)	42(45%)
Strangulation	32 (48%)	8 (38%)	1 (17%)	41(44%)
Volvulus	3 (5%)	1 (5%)	0	4 (4%)
Other	6 (9%)	0	0	6 (6%)
Bowel additionally preserved/resected in cm, median (IQR)	n.a.	50 (28–98)	20 (6–50)	n.a.
Reoperation rate	5 (8%)	6 (29%)	0	11 (12%)
Planned reoperations	1 (2%)	4 (19%)	0	5 (5%)
Unplanned reoperations	4 (7%)	2 (10%)	0	6 (6%)
Mortality	10 (15%)	5 (24%)	2 (33%)	17 (18%)

### Reoperations

The overall 30-day reoperation rate was 12% (*n* = 11) with four patients having progressive ischaemia (Table 3). In the group without a CoM-FA (*n* = 66/93), there was a reoperation rate of 8% (*n* = 5/66), comprising four unplanned reoperations and one planned second look. One patient in this group had further bowel ischaemia identified (one of the four unplanned reoperations). In the CoM-FA group, 22% (*n* = 6/27) of the patients had a reoperation, all concerning patients with a more conservative approach after FA. Four planned (second look) surgeries (two of whom had further bowel ischaemia needing resection) and two unplanned operations (of whom one had further ischaemia). In patients who did not undergo a resection, 5% (*n* = 2/43) needed a reoperation because of progressive ischaemia. Reoperation rates were 20% (*n* = 8/41) for consultants and 6% (*n* = 3/52) for senior surgical trainees. Regarding the surgical approach, 15% (*n* = 10/66) of laparotomies and 4% (*n* = 1/20) of laparoscopies required reoperation. An anastomotic leak occurred in one out of 24 patients (4%) with an anastomosis with the diagnosis being made during a planned second-look surgery.

**Table 3:** Reoperations specified per patient group

Patient group	Unplanned reoperation	Planned reoperation (second look)
No CoM-FA (*n* = 5/66)	1. Intra-abdominal bleeding query, no bleeding found 2. Intra-abdominal bleeding query, additional resection of ischaemic cecal pole 3. Intra-abdominal lavage because of infected hematoma 4. Intra-abdominal lavage because of infected hematoma	1. Restoration of bowel continuity
CoM-FA		
More conservative approach (*n* = 6/27)	1. Evisceration and intra-peritoneal mesh placement 2. Progressive ischaemia: 30 cm additional bowel resected	1. Negative second look 2. Restoration of bowel continuity 3. Progressive ischaemia: additional resection of 50 cm small bowel 4. Progressive ischaemia: additional resection of 230 cm small bowel
More aggressive approach (*n* = 0/6)	n.a.	n.a.

### Mortality

In total, 17 patients died in this cohort, with an overall 30-day mortality rate of 18% (Table 2). Mortality rates were 15% (*n* = 10/66) in the no CoM-FA group, 24% (*n* = 5/21) in the group with a more conservative approach and 33% (*n* = 2/6) with a more aggressive approach. In 76% (*n* = 13/17) of patients who died, the underlying aetiology was mesenteric ischaemia and in 24% (*n* = 4/17) strangulation. Among the patients who died, 12 died due to sepsis and multi organ failure. Other causes of death were pneumonia (*n* = 2), cardiac arrest (*n* = 1) and liver failure (*n* = 1), acute haemorrhage after a thrombectomy of the superior mesenteric artery (*n* = 1). The mortality rate was higher in the laparotomy group: 23% (*n* = 15/66), compared to the laparoscopic or conversion group (5%, *n* = 1/20), 14% (*n* = 1/7), respectively. Among surgeries performed by consultants mortality rates were 27% (*n* = 11/41) compared to 12% (*n* = 6/52) performed by senior surgical trainees.

## Discussion

This international, multi-centre cohort study describes the use and outcomes of FA in the acute setting. Although FA has been widely implemented in the clinical setting for elective surgery, the potential added value in the acute setting has barely been studied. Among 93 operations for acute bowel ischaemia, a change of management was observed in 29%, resulting in bowel preservation in approximately one out of four patients without a substantial increase in unplanned reoperations. The overall reoperation rate was 12% and the 30-day mortality was 18%, both of which are low compared to other published series [1–4, 13]. In half of the patients that underwent bowel resection, a primary anastomosis was made. In these patients, an acceptable leak rate of 4% was found.

This study also emphasizes that a median of 50 cm (IQR 28–98) of bowel could be spared among patients with a CoM-FA with a more conservative approach. Preserving 50 cm of small bowel or colon could make the difference in patients with extensive resections in preventing subsequent short bowel syndrome (which tends to occur when less of 100 cm of functioning bowel remains) [14].

The number of CoM-FA reported in this study corresponds with two prior studies investigating the use of FA in the acute setting; in both studies, FA provided additional information in 32–34.6% of the cases [15, 16]. While definitive randomized control trials are currently ongoing [17], existing literature indicates that ICG use in vascularization assessment impacts the user’s decision making in the elective setting in approximately 5–15% of cases [18, 19]. It seems therefore that FA assessments alter the surgical strategy more often in the acute than in the elective setting.

In our study, there was an overall reoperation rate of 12%. This was 29% in patients in whom the CoM- FA encouraged preservation of bowel versus 8% in those in whom there was no CoM-FA. It’s concerning that three patients in this group had further ischaemia identified and the mortality of this group was also higher than those in whom either no change or a more aggressive approach was followed. Some of the increased reoperation rate may be due to surgeons planning a second look with a lower threshold or to perform a delayed anastomosis after a more conservative approach is conceivable. Unplanned operation rates were similar between those without CoM-FA (7%) as with those in whom CoM-FA preserved bowel (10%) and in both groups, one patient was found to have progressive ischaemia. Three of the four cases requiring additional resection, however, took place in the conservative group, indicating that FA interpretation could be misleading and might require a proper learning curve to be proficient. As acute surgery is often performed out of office hours, the surgeon on call might have less experience using FA and interpret the FA differently than a more experienced user would, it has recently been demonstrated that there is a significant inter-observer variability of the interpretation of fluorescence imaging between expert and non-expert users in the elective setting [20]. Besides, in the acute setting, the surgeon has to contend with haemodynamic unstable patients with vasopressor requirement, which might result in difficult to interpret fluorescence images. When preserving bowel due to FA, there does seem to be a higher risk of progressive ischaemia and performing a planned reoperation (second look) at a lower threshold should be considered when preserving bowel.

The overall 30-day mortality was 18% (*n* = 17/93) in this series. If one looks at the mortality rates within the CoM-FA groups (Table 2), there is a higher mortality rate (24% in conservative group and 33% in the more aggressive approach group), compared to the no CoM-FA group (15%). This is noteworthy; however, this does not necessarily mean that this is related to the change of management itself. The CoM-FA groups have a different aetiology than the no change group, i.e. a higher proportion of mesenteric ischaemia, which is associated with a higher morbidity and mortality [3, 4]. Besides, in the CoM-FA group, there was a higher proportion of patients with an ASA score of IV; 44% versus 21% (Table 1). Also, it may be those who had CoM-FA had more complex ischaemic patterns to judge while those without were more discrete. It's noteworthy that consultant-case had higher mortality overall then those done by more junior staff, similarly suggesting differences other than this parameter alone.

While FA seems associated with positive outcomes in the overall group, the concerns identified in this study accentuate the need for further careful research including perhaps data-banking of the videos to allow understanding of the perfusion appearances and user interpretations. For instance, after reviewing FA images of patients who had progressive ischaemia during reoperation, two videos gave the impression of diffusion rather than adequate perfusion in the more conservative group and in our opinion should have been resected initially. This would also allow for training of surgeons without each having to acquire individual expert experience on the job. Furthermore quantification methods, with quantitative values that define a threshold for adequate perfusion, may help standardize interpretations. In addition, protocolized approaches are needed to be developed. From our own experience, endorsed by this study, we suggest that perfusion judgement should be determined within the first minute as, even though the bowel may fluoresce later, this may be falsely positive occurring due to diffusion of ICG into the tissue rather than true perfusion [21].

This study was limited by its retrospective nature. However, CoM-FA was reported in the operation reports prospectively. Yet, due to the retrospective nature of this study, we could not distinguish between the different kinds of aetiology of non-adhesional and non-volvulus bowel ischaemia (i.e. whether low flow, thrombotic disease, non-occlusive mesenteric ischaemia). In addition, quantitative values such as time to fluorescence were not captured. Besides, patients were not consecutively included, which may have resulted in a selection bias. There is also a lack of contemporaneous control data to give context to the clinical outcomes here reported. Prospective studies which both include quantitative fluorescence values as well as differentiate between the different manifestations of intestinal ischaemia are needed.

In conclusion, this study, the largest multi-centre case series yet, presents the use of FA in the acute setting of patients operated on because of ischaemia. Results from this study support that FA can provide useful, additional information besides visual evaluation alone on a reproducible manner. However, prospective studies are needed to optimize the best use of this technology for this indication and to determine standards for the interpretation of FA images and the potential subsequent need for second-look surgeries.

## Supplementary Information

Below is the link to the electronic supplementary material.

Supplementary file1 (DOCX 13 kb)
