# Supplementary material for: The use of fluorescence angiography to assess bowel viability in the acute setting: an international, multi-centre case series
Source: Surg Endosc. 2022 Feb 23;36(10):7369–75. doi: 10.1007/s00464-022-09136-7 (PMC9485089; doi:10.1007/s00464-022-09136-7)
Supplement: Supplementary file 1 — Supplementary file1 (DOCX 13 kb) [file 464_2022_9136_MOESM1_ESM.docx]

**Supplementary Table 1.** Overview of the changes in bowel length preserved and additionally resected for CoM-FA groups

| **CoM-FA: more conservative approach** | | | **CoM-FA: more aggressive approach** | | |
| --- | --- | --- | --- | --- | --- |
| Patient | Specifications bowel | Amount(cm) | Patient | Specifications bowel | Amount(cm) |
| 1. | no resection: small bowel preserved | 30 | 1. | colon and small bowel | 50 |
| 2. | small bowel | 25 | 2. | colon and small bowel | 10 |
| 3. | small bowel | 60 | 3. | colon and small bowel | 2 |
| 4. | small bowel | 5 | 4. | small bowel | 50 |
| 5. | small bowel | 50 | 5. | small bowel | 20 |
| 6. | small bowel | 70 | 6. | small bowel | 20 |
| 7. | small bowel | 10 |  |  |  |
| 8. | colon and small bowel | 170 |  |  |  |
| 9. | colon and small bowel | 5 |  |  |  |
| 10. | colon and small bowel | 50 |  |  |  |
| 11 | colon and small bowel | 170 |  |  |  |
| 12. | small bowel | 50 |  |  |  |
| 13. | small bowel | 95 |  |  |  |
| 14. | small bowel | 50 |  |  |  |
| 15. | small bowel | 100 |  |  |  |
| 16. | small bowel | 40 |  |  |  |
| 17. | small bowel | 200 |  |  |  |
| 18. | colon and small bowel | 100 |  |  |  |
| 19. | no resection: small bowel preserved | 70 |  |  |  |
| 20. | no resection: small bowel preserved | 20 |  |  |  |
| 21. | no resection: small bowel preserved | 50 |  |  |  |
